# Supplementary material for: Iterative improvement in the automatic modular design of robot swarms
Source: PeerJ Comput Sci. 2020 Dec 7;6:e322. doi: 10.7717/peerj-cs.322 (PMC7924708; doi:10.7717/peerj-cs.322)
Supplement: Supplemental Information 3 [file peerj-cs-06-322-s003.zip › argos3/doc/api/standalone/a00547_source.html]

ARGoS: plugins/robots/mini-quadrotor/control\_interface/ci\_miniquadrotor\_rotor\_actuator.h Source File


- Main Page
- Related Pages
- Namespaces
- Classes
- Files

- File List
- File Members

# plugins/robots/mini-quadrotor/control\_interface/ci\_miniquadrotor\_rotor\_actuator.h

Go to the documentation of this file.

```
00001 
00007 #ifndef MINIQUADROTOR_ROTOR_ACTUATOR_H
00008 #define MINIQUADROTOR_ROTOR_ACTUATOR_H
00009 
00010 namespace argos {
00011    class CCI_MiniQuadrotorRotorActuator;
00012 }
00013 
00014 #include <argos3/core/control_interface/ci_actuator.h>
00015 
00016 namespace argos {
00017 
00018    class CCI_MiniQuadrotorRotorActuator : public CCI_Actuator {
00019 
00020    public:
00021 
00022       struct SVelocities {
00023          Real Velocities[4];
00024 
00025          SVelocities();
00026          SVelocities(Real f_north_vel,
00027                      Real f_west_vel,
00028                      Real f_south_vel,
00029                      Real f_east_vel);
00030          SVelocities(const SVelocities& s_velocities);
00031          SVelocities& operator=(const SVelocities& s_velocities);
00032       };
00033 
00034    public:
00035 
00036       virtual ~CCI_MiniQuadrotorRotorActuator() {}
00037 
00038       virtual void SetRotorVelocities(const SVelocities& s_velocities) = 0;
00039 
00040 #ifdef ARGOS_WITH_LUA
00041       virtual void CreateLuaState(lua_State* pt_lua_state);
00042 #endif
00043 
00044    protected:
00045 
00046       SVelocities m_sCurrentVelocities;
00047 
00048    };
00049 
00050 }
00051 
00052 #endif
```

---

Generated on 10 Jul 2018 for ARGoS by 
 1.6.1
